# Insulin resistance, lipids and body composition in patients with coronary artery disease after combined aerobic training and resistance training: a randomised, controlled trial

**DOI:** 10.1186/s13098-023-01017-w

**Published:** 2023-03-15

**Authors:** Tim Kambic, Mojca Božič Mijovski, Borut Jug, Vedran Hadžić, Mitja Lainscak

**Affiliations:** 1 Cardiac Rehabilitation Unit, General Hospital Murska Sobota, 9000 Murska Sobota, Slovenia; 2 Faculty of Sport, University of Ljubljana, 1000 Ljubljana, Slovenia; 3 Laboratory for Haemostasis and Atherothrombosis, Department of Vascular Diseases, University Medical Centre Ljubljana, 1000 Ljubljana, Slovenia; 4 Faculty of Pharmacy, University of Ljubljana, 1000 Ljubljana, Slovenia; 5 Department of Vascular Diseases, University Clinical Centre Ljubljana, 1000 Ljubljana, Slovenia; 6 Faculty of Medicine, University of Ljubljana, 1000 Ljubljana, Slovenia; 7 Division of Cardiology, Department of Internal Medicine, General Hospital Murska Sobota, Rakican, Ulica dr. Vrbnjaka 6, 9000 Murska Sobota, Slovenia

**Keywords:** Insulin sensitivity, Blood lipids, Cardiac rehabilitation, Acute coronary syndrome.

## Abstract

**Background:**

The effect of resistance training (RT) in cardiac rehabilitation (CR) on insulin resistance remains elusive. We examined whether the addition of high-load (HL) or low loads (LL) RT has any effect on the levels of insulin resistance and lipids versus aerobic training (AT) alone in patients with coronary artery disease (CAD).

**Methods:**

Seventy-nine CAD patients were randomised to HL-RT [70–80% of one repetition maximum (1-RM)] and AT, LL-RT (35–40% of 1-RM) and AT or AT (50–80% of maximal power output), and 59 patients [75% males, 15% diabetics, age: 61 (8) years, left ventricular ejection fraction: 53 (9) %] completed the study. Plasma levels of glucose, insulin, blood lipids [total cholesterol, triglycerides, high-density lipoprotein (HDL) cholesterol and low-density lipoprotein (LDL)] cholesterol and body composition were measured at baseline and post-training (36 training sessions).

**Results:**

Training intervention had only time effect on lean mass (p = 0.002), total and LDL cholesterol levels (both p < 0.001), and no effects on levels of glucose and insulin resistance (homeostatic assessment 2-insulin resistance). Total and LDL cholesterols levels decreased following AT [mean difference (95% confidence interval); total cholesterol: − 0.4 mmol/l (− 0.7 mmol/l, − 0.1 mmol/l), p = 0.013; LDL: − 0.4 mmol/l (− 0.7 mmol/l, − 0.1 mmol/l), p = 0.006] and HL-RT [total cholesterol: − 0.5 mmol/l (− 0.8 mmol/l, − 0.2 mmol/l), p = 0.002; LDL: − 0.5 mol/l (− 0.7 mmol/l, − 0.2 mmol/l), p = 0.002]. No associations were observed between post-training change in body composition and post-training change in blood biomarkers.

**Conclusions:**

RT when combined with AT had no additional effect beyond AT alone on fasting glucose metabolism, blood lipids and body composition in patients with CAD.

*Trial registration number* NCT04638764.

**Supplementary Information:**

The online version contains supplementary material available at 10.1186/s13098-023-01017-w.

## Background

Exercise training is a core component of cardiac rehabilitation (CR) [1, 2] and has been associated with improvements of physical performance, body composition and quality of life, as well as blood pressure, glucose metabolism and lipid control [1, 3, 4]. While the effects of multicomponent exercise-based CR on physical performance, body composition and quality of life are evident [5–7], less is known about CR effects on insulin resistance and lipids in patients with CAD [4], despite high prevalence of diabetes and dysmetabolism (54%) among patients enrolled in an early phase II CR [8].

During the early stage of phase II CR, the standard care is focused mainly on clinical assessments of cardiac function and risk factors, and optimisation of pharmacological therapy [9], while less emphasis is given on the initial implementation of progressive training programmes with optimal training loading due to the lack of exact training recommendation [10], which would otherwise greatly improve the efficacy of CR efficacy. Therefore, previous studies in patients with CAD have applied only low-load (LL) to moderate-load RT [50–65% of one repetition maximum (1-RM)] in combination with moderate to high intensity AT and mostly showed no additional benefits on glucose metabolism and blood lipids when compared with control [11, 12] and/or AT alone [12, 13]. Whilst only the superior effects on maximal muscle strength were established following combined AT with high-load (HL) in our recent study [14], such efficacy over combined AT with LL-RT or AT alone remains to be established on insulin resistance and lipids.

Since the recent recommendations for patients with CAD and coexisting diabetes advocates for the use of combined AT and RT at the highest intensity possible to achieve optimal control of glucose metabolism, dyslipidaemia and body composition in early phase of CR [9], our study aimed to determine whether the dose-dependent relationship between RT load (LL-RT vs HL-RT) and improvements of glucose metabolism and lipids profile exists in patients with CAD.

## Methods

### Study design

**Fig. 1:**
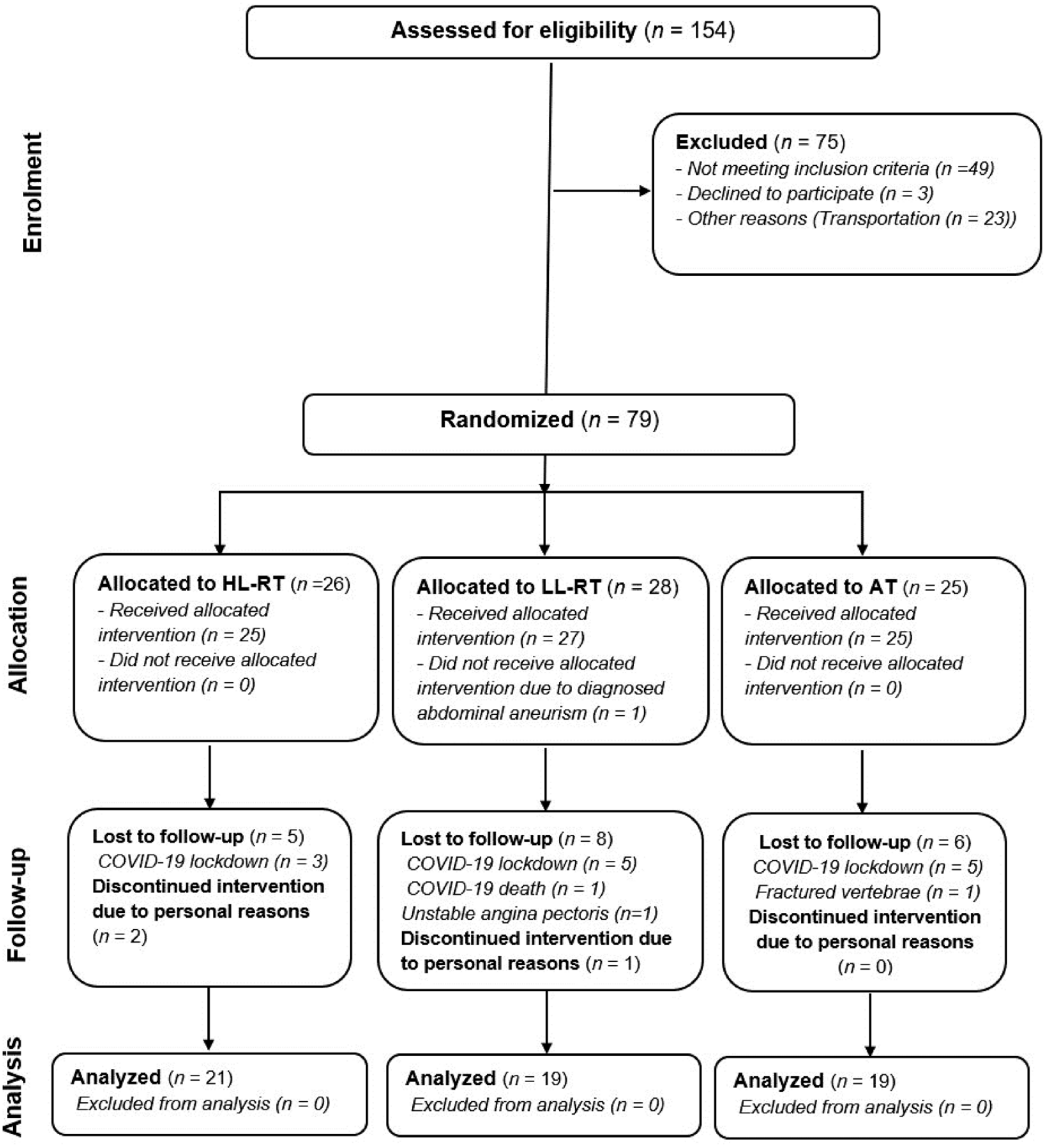
CONSORT flow chart of the study. *HL-RT* high-load resistance training, *LL-RT* low loads resistance training, *AT* aerobic training, *COVID-19* Coronavirus disease-19

This study presents a prespecified secondary analysis of a randomised controlled trial (ClinicalTrials.gov Identifier: NCT04638764). Patients with CAD were cluster randomised to three parallel training interventions (Fig. 1): combination of HL-RT with AT; combination of LL-RT with AT; and solely AT as a standard care. The study was designed in accordance with the CONSORT guidelines [15] and Declaration of Helsinki, and was approved by the National Medical Ethics Committee (registration number: 0120-573/2019/15). The study protocol, the feasibility and safety, and the primary outcomes of the study were published previously [14, 16, 17].

The outcomes of this secondary analysis of the randomised controlled trial were: change in glucose, insulin and insulin resistance [homeostatic model assessments of insulin resistance (HOMA-IR)], blood lipids (cholesterol, HDL, LDL and triglycerides), and body composition (body weight, lean mass and fat mass), following training intervention. The assessor of the study was not blinded to group allocation due to COVID-19 outbreak staff reassignments.

Patients were assessed at baseline and post-training (> 24–36 training sessions). Body composition was measured at baseline (7–10 days prior to enrolment) and post-training (3–7 days after last training session) to exclude potential false muscle hypertrophy due to acute muscle swelling post last RT session. Blood samples were collected after overnight fast (≥ 10 h) in the morning prior to first and last training sessions (48–72 h after the last session) In addition, maximal aerobic capacity and lower limb muscle strength were assessed at baseline and after seven weeks (only maximal leg press strength) to determine AT and RT workloads.

### Study sample

The study recruited patients with CAD (acute coronary syndrome and/or percutaneous coronary intervention) from the Division of Cardiology, General Hospital Murska Sobota, Slovenia. Inclusion criteria were age (18–85 years), left ventricular ejection fraction ≥ 40%, documented CAD (≥ 1 month after clinical event), referral to phase II outpatient CR, and completion of a baseline cardiopulmonary exercise test [2]. Exclusion criteria were aligned with standard recommendations for participation in RT [4, 18]. Prior to enrolment, all patients received verbal and written information about the study aims, procedures and potential risk during the study and were asked to sign a written informed consent before beginning study procedures.

### Training protocol

Training intervention was embedded in a standard phase II out-patient CR consisted of three weekly training sessions for 12 weeks (i.e., 36 training sessions), with 48–72 h rest between sessions.

All patients performed aerobic interval cycling (3–5 min workload cycling/2 min unloaded cycling, a total of 40 min/session) starting from the initial 50% of maximal workload achieved at baseline cardiopulmonary exercise test and progressively increasing every two weeks to 80% maximal workload [17]. Cycling cadence was set at 50–60 revolutions per min [2].

Patients randomised to RT completed a total of 36 sessions on a leg-press machine (three 1-RM tests and 33 RT sessions). In HL-RT group the workload was increased from an initial three sets at intensity 70% of 1-RM (6–11 repetitions/set) to 80% of 1-RM (6–8 repetitions per set) in the first seven weeks of the CR, while the workload in the LL-RT group increased from the initial 35% of 1-RM (12–22 repetitions/set) to 40% of 1-RM (12–16 repetitions per set). Similar progression in both RT groups was applied following 1-RM re-evaluation after 7 weeks of training [17, 19–21]. A lifting cadence of 1 s: 1 s (concentric and eccentric contraction) was used, with 90 s rest between sets [22]. Each RT session lasted for 7–10 min and was performed between intervals of unloading intervals of AT in a changing, randomised order for all patients in each training group to eliminate potential effects of fatigue. The entire study protocol is available elsewhere [17].

## Measures

### Maximal aerobic capacity

Maximal aerobic capacity was measured using an adjusted ramp protocol [23] on a Schiller ERG 911 ergometer and using mask connected to a breath-by-breath Cardiovit CS-200 Excellence ErgoSpiro system (Schiller, Baar, Switzerland). Patients were first instructed and familiarised with correct breathing technique followed by a spirometry test. Afterwards, patients remained seated for determination of baseline gas exchange and hemodynamic (heart rate and blood pressure) values. Maximal aerobic capacity was assessed using adjusted ramp protocol by increasing workload every minute for an additional 10–25 W until exhaustion or other contraindication [17, 23].

### Maximal leg strength

Submaximal strength test assessments and RT were performed using a Life Fitness Leg Press Pro 2 (Life Fitness Inc., Rosemont, Illinois, USA). Patients were first familiarised with correct lifting and breathing technique, which was followed by two warm-up sets comprising of eight and six repetitions at 50% and 70% of patients` perceived 1-RM, respectively. Afterwards, the weight was progressively increased until reaching the workload that could be lifted three to five times (3–5 RM). Trials were separated with a two to three min rest [20]. The 1-RM was calculated using the established 1-RM prediction equation (predicted 1-RM = maximal load lifted/1.0278–0.0278 × number of repetitions) [24].

### Blood biomarkers

Blood samples were drawn from the right antecubital vein using 21-gauge needle (40 mm) into 2,5 mL and 10 mL BD Vacutainer^®^ vacuum serum tube with silica particles coating (Becton, Dickinson and Company, Vacutainer System Europe, Heidelberg, Germany). Serum samples were prepared with 10-min centrifugation at 2700 rpm and 20 °C using Eppendorf 5810 R centrifuge (Eppendorf Ag, Hamburg, Germany). After centrifugation, 2,5 mL serum tubes were immediately used for analysis of glucose, triglycerides, total cholesterol, high-density lipoprotein [HDL] cholesterol and low-density lipoprotein [LDL] cholesterol concentrations using Roche Cobas 8000–1 modular analyser (Roche Diagnostics Ltd., Rotkreuz, Switzerland). From 10 mL vacuum tubes serum was aliquoted into 1, 8 mL Sarstedt cryovials (Sarstedt Ag and Company, Nümbrecht, Germany) and stored ≤ − 70 °C within two hours until further analysis of insulin levels. Insulin levels were measured in a thawed serum aliquote with the Luminex’s xMAP^®^ technology utilizing magnetic beads coupled with specific antibodies, with allowed multiplexing. Analysis was performed using a MagPix analyzer in line with manufacturer’s instructions (all R&D Systems, Abingdon, United Kingdom). Homeostatic model assessment 2 of insulin resistance (HOMA2-IR) was calculated using values glucose and insulin levels using well established HOMA2 equation [25] and calculator.^1^

### Anthropometry and body composition

Body height and mass were measured on Marsden DP3810 weighing scale and stadiometer (Marsden Weighing Group, Rotherham, UK), and body lean mass and body fat mass were measured using bioimpedance measurement with a Bodystat Quadscan 4000 Touch (Bodystat, Douglas, Isle of Man, UK). Measurement of body composition was performed in the morning (before 10 a.m.) in line with reported protocol [17]. Post-training measurements were performed during the same time of the day as were at baseline (± 2 h).

### Statistical analysis

Sample size was calculated for primary outcomes [maximal aerobic capacity (ml/kg/min) and maximal voluntary contraction (Nm)], and the exact calculations were previously published [14, 17]. This study presents pre-specified secondary outcomes of body composition, glucose and insulin metabolism and blood lipids, which should be only interpreted as exploratory.

Data are presented as numbers and percentages for descriptive variables and as means (standard deviations) or medians (interquartile ranges) (according to the normality of distribution) for numeric variables. Numeric variables were screened for normality of distribution (Shapiro–Wilk test), homogeneity of variances (Levene test) and sphericity (Mauchly test). Data were analysed using per-protocol analysis [17], and we included all patients who completed > 24 training sessions in the final analysis. Between-group differences in baseline were assessed using one-way analysis of variance (ANOVA) or the Kruskall–Wallis test (depending on the assumptions), with additional post-hoc analysis using Tukey or Bonferroni tests. Training effect was assessed using two-way repeated measures ANOVA or analysis of covariance (ANCOVA), in case of significant baseline difference between groups. The within-group training effect was calculated using Bonferroni adjustment for multiple comparisons within two-way ANOVA. In addition to ANCOVA, paired sample t-tests or Wilcoxon tests was used, accordingly, to assess within-group improvement following training. The reported effect size is partial eta squared (η^2^). Comparison between training groups in categorical outcomes was calculated using Chi-square test or Fisher exact test. Correlation between post-training difference (post-training difference = post-training-baseline value) in body composition and blood biomarkers was calculated using Spearman rank correlation coefficient. Comparison between baseline and post-training values of blood markers in patients with and without diabetes was calculated using paired-samples t-test or Wilcoxon rank test. IBM SPSS 25 software (SPSS Inc., Armonk, NY, USA) was used for the analysis at a level of statistical significance set at alpha < 0.05.

## Results

One hundred and fifty-four patients with CAD were screened for eligibility and 79 were included in the study (Fig. 1). During the study, 20 patients were lost to follow-up, mainly due to personal and medical reasons, and 59 patients were included in the final per-protocol analysis. On average, patients were 61 (8) (years old, had left ventricular ejection fraction 53(9) %), and were mostly males (75%) and non-smokers or ex-smokers (83%). In the AT group, more patients were diagnosed with atrial fibrillation than in the HL-RT and LL-RT groups (*p* = 0.038). Otherwise, there no between-group differences in baseline anthropometry, clinical characteristics, smoking status and pharmacological therapy (Table 1). Following the training intervention, the dose of statins or ezetimibe significantly increased in all three training groups (AT: + 7 mg, p = 0.010; LL-RT: + 7 mg, p = 0.023; HL-RT: + 11 mg, p < 0.001), with no significant time x group interaction (Additional file 1: Table S1). There was also no difference between training groups in lipid lowering drug at baseline (p = 0.836) and post-training (p = 0.426) (Additional file 1: Table S2). Training adherence AT and RT was very high, only eight patients completed less than 36 AT sessions (one patient completed 35 sessions in AT group; one patient completed 34 sessions and four patients completed 35 sessions in LL-RT group; two patients completed 35 sessions in HL-RT group), and only one patient failed to complete all HL-RT sessions (35 completed sessions).

**Table 1:** Anthropometry, clinical characteristics and cardiovascular risk factors at baseline

Variable	Sample (n = 59)	AT group (n = 19)	LL-RT group (n = 19)	HL-RT group (n = 21)	*p*
Age (years)	61 (8)	61 (9)	61 (7)	62 (8)	0.910
Sex [males, (%)]	44 (75)	14 (74)	15 (79)	15 (71)	0.931
Anthropometry					
Height (cm)	172.1 (8.4)	170.4 (8.8)	172.8 (8.6)	172.9 (7.9)	0.582
Weight (kg)	85.47 (15.43)	90.94 (19.04)	81.46 (13.37)	84.15 (12.56)	0.148
Clinical data					
LVEF (%)	53 (9)	50 (45,60)	55 (50, 60)	50 (45,58)	0.454
Time from clinical event to inclusion in CR (months)	2.0 (1.5, 3.0)	2.0 (2.0,2.5)	2.5 (1.5, 3.0)	2.0 (1.5, 2.8)	0.832
Myocardial infarction, f (%)					
NSTEMI	25 (42)	9 (47)	8 (42)	8 (38)	0.947
STEMI	24 (41)	7 (37)	7 (37)	10 (48)	
Unstable AP/PCI	10 (17)	3 (16)	4 (21)	3 (14)	
Comorbidities and risk factors, f (%)					
Arterial hypertension	41 (70)	15 (79)	11 (58)	15 (71)	0.383
Hyperlipidemia	49 (83)	16 (84)	14 (74)	19 (91)	0.384
Diabetes	9 (15)	4 (21)	3 (16)	2 (10)	0.602
Atrial fibrillation	5 (9)	4 (21)	1 (5)	0 (0)	0.038
Thyroid disease	5 (9)	2 (11)	2 (11)	1 (5)	0.727
Renal disease	4 (7)	0 (0)	2 (11)	2 (10)	0.534
Smoking, f (%)					
Non-smoker	14 (24)	3 (16)	3 (16)	8 (38)	0.346
Ex-smoker	35 (59)	13 (68)	11 (58)	11 (52)	
Current smoker	10 (17)	3 (16)	5 (26)	2 (10)	
Pharmacological therapy, f (%)					
ASA	57 (97)	17 (90)	19 (100)	21 (100)	0.200
Beta blocker	59 (100)	19 (100)	19 (100)	21 (100)	1.000
ACE inhibitor/ARB	58 (98)	19 (100)	18 (95)	21 (100)	0.644
Statin/Ezetimibe	59 (100)	19 (100)	19 (100)	21 (100)	1.000
Antiplatelets	58 (98)	18 (95)	19 (100)	21 (100)	0.644
Anticoagulation	5 (9)	3 (16)	1 (5)	1 (5)	0.509
Diuretic	5 (9)	4 (21)	0 (0)	1 (5)	0.071

Data are presented as mean (standard deviation) or as median (first quartile, third quartile)

*AT* aerobic training, *LL-RT* low-load resistance training, *HL-RT* high-load resistance training, *LVEF* left ventricular ejection fraction, *(N)STEMI* (non)ST-segment-elevated myocardial infarction, *AP* angina pectoris, *PCI* percutaneous coronary intervention, *ASA* acetylsalicylic acid, *ACE* angiotensin-converting enzyme, *ARB* angiotensin II receptor blockers

With exception of significant difference between groups in baseline triglycerides (p = 0.014), training groups did not differ in baseline glucose and insulin levels, HOMA2-IR and other blood lipids (Table 2). After adjusting for baseline difference, there was no significant difference between groups in post-training triglycerides levels (p = 0.927). Two-way ANOVA has shown a significant effect of time on total cholesterol and LDL (both p < 0.001), but no effects of time x group interaction on glucose levels, insulin levels, HOMA2-IR and blood lipids (all interaction p > 0.326). When compared with baseline, total cholesterol and LDL were significantly lower following AT [total cholesterol: − 0.4 mmol/l (− 0.7 mmol/l, − 0.1 mmol/l), p = 0.013; LDL: − 0.4 mmol/l (-0.7 mmol/l, − 0.1 mmol/l), p = 0.006] and HL-RT [total cholesterol: − 0.5 mmol/l (− 0.8 mmol/l, − 0.2 mmol/l), p = 0.002; LDL: − 0.5 mol/l (− 0.7 mmol/l, − 0.2 mmol/l), p = 0.002].

**Table 2:** Baseline and post training levels of glucose, insulin resistance and blood lipids

Blood marker	Group	N	Baseline	Post-training	2-way ANOVA/**ANCOVA**	
					Time effect/**effect of baseline**	Interaction/**post-training difference**
Glucose (mmol/l)	AT	15	6.0 (1.2)	6.1 (1.4)	p = 0.741 η^2^ = 0.002	p = 0.791 η^2^ = 0.010
	LL-RT	16	5.6 (0.6)	5.5 (0.7)		
	HL-RT	19	5.6 (0.5)	5.7 (0.5)		
Insulin (pmol/l)	AT	15	95 (46)	98 (58)	p = 0.923 η^2^ = 0.000	p = 0.885 η^2^ = 0.005
	LL-RT	16	78 (38)	77 (31)		
	HL-RT	19	74 (56)	70 (44)		
HOMA2-IR (units)	AT	15	1.82 (0.86)	1.90 (1.12)	p = 0.965 η^2^ = 0.000	p = 0.880 η^2^ = 0.005
	LL-RT	16	1.49 (0.71)	1.46 (0.58)		
	HL-RT	19	1.40 (1.05)	1.34 (0.82)		
Total cholesterol (mmol/l)	AT	19	3.8 (1.1)	3.4 (0.9)	p < 0.001 η^2^ = 0.013	p = 0.492 η^2^ = 0.025
	LL-RT	19	3.2 (0.7)	3.0 (0.5)		
	HL-RT	21	3.6 (0.9)	3.2 (0.5)		
HDL (mmol/l)	AT	19	1.2 (0.5)	1.3 (0.4)	p = 0.961 η^2^ = 0.000	p = 0.573 η^2^ = 0.020
	LL-RT	19	1.2 (0.4)	1.2 (0.3)		
	HL-RT	21	1.2 (0.3)	1.2 (0.3)		
LDL (mmol/l)	AT	19	2.0 (1.0)	1.6 (0.7)	p < 0.001 η^2^ = 0.260	p = 0.499 η^2^ = 0.025
	LL-RT	19	1.6 (0.5)	1.4 (0.4)		
	HL-RT	21	2.0 (0.7)	1.5 (0.4)		
Triglycerides (mmol/l)	AT	19	1.8 (1.0)	1.7 (0.9)	**p < 0.001** **η**^**2**^** = 0.649**	**p = 0.927** **η**^**2**^** = 0.003**
	LL-RT	19	1.3 (0.4)	1.3 (0.5)		
	HL-RT	21	1.3 (0.4)	1.2 (0.4)		

Data are presented as mean (standard deviation) or as median (first quartile, third quartile). Text in bold presents ANCOVA results. Glucose, insulin and HOMA2-IR are analysed only for nondiabetic patients

*HOMA2-IR* homeostatic model assessment for insulin resistance, *HDL* high density lipoprotein, *LDL* low density lipoprotein, *AT* aerobic training, *LL-RT* low-load resistance training, *HL-RT* high-load resistance training, *ANOVA* analysis of variance, *ANCOVA* analysis of covariance, *η*^*2*^ partial eta squared (effect size)

Table 3 presents the change of body mass, lean mass and fat mass following training intervention in all groups. Training groups significantly differed in baseline fat mass (LL-RT vs AT = − 8.20 kg, p = 0.035). After adjusting for baseline difference, there was no significant differences between groups in post-training fat mass. Two-way repeated measures ANOVA has shown a significant time effect for lean mass, but no effects of time x group interaction on any of the body composition variables. When compared with baseline, AT group significantly increased fat % [mean difference (95% Confidence interval for mean difference), + 1% (0%, + 2%), p = 0.048], decreased lean mass % [− 1% (0%, − 2%), p = 0.048] and lean mass [− 1.05 kg (− 1.89 kg, − 0.20 kg), p = 0.016] following the training intervention. Similarly, HL-RT group significantly decreased lean mass [− 1.05 kg (− 1.87 kg, − 0.22 kg), p = 0.014].

**Table 3:** Baseline and post-training body composition

Body composition measure		N	Baseline	Post-training	2-way ANOVA/**ANCOVA**	
					Time effect/**effect of baseline**	Interaction/**post-training difference**
Body mass (kg)	AT	19	90.94 (19.04)	90.49 (17.87)	p = 0.187 η^2^ = 0.031	p = 0.974 η^2^ = 0.001
	LL-RT	19	81.46 (13.37)	80.91 (13.90)		
	HL-RT	21	84.15 (12.56)	83.47 (13.48)		
Fat (%)	AT	19	28.2 (9.2)	29.2 (8.7)	p = 0.500 η^2^ = 0.008	p = 0.138 η^2^ = 0.070
	LL-RT	19	22.3 (4.7)	22.0 (5.2)		
	HL-RT	20	24.9 (8.4)	24.7 (7.6)		
Fat (kg)	AT	19	26.0 (11.0)	26.7 (10.3)	**p < 0.001** **η**^**2**^** = 0.900**	**p = 0.095** **η**^**2**^** = 0.083**
	LL-RT	19	17.8 (3.3)	17.6 (4.5)		
	HL-RT	20	21.0 (8.3)	20.5 (7.5)		
Lean (%)	AT	19	71.8 (9.2)	70.8 (8.7)	p = 0.497 η^2^ = 0.008	p = 0.139 η^2^ = 0.069
	LL-RT	19	77.7 (4.7)	78.0 (5.2)		
	HL-RT	20	75.1 (8.4)	75.3 (7.6)		
Lean (kg)	AT	19	64.9 (13.6)	63.9 (13.1)	p = 0.002 η^2^ = 0.166	p = 0.354 η^2^ = 0.037
	LL-RT	19	63.6 (12.5)	63.3 (12.4)		
	HL-RT	20	63.4 (11.7)	62.4 (11.5)		

Data are presented as mean (standard deviation) or as median (first quartile, third quartile). Text in bold presents ANCOVA results

*LL-RT* low load resistance training, *HL-RT* high load resistance training, *AT* aerobic training, *ANOVA* analysis of variance, *ANCOVA* analysis of covariance, *η*^*2*^ partial eta squared (effect size)

Additional exploratory analysis of associations between post-training difference in blood markers and post-training difference body composition revealed no significant correlation when calculated on a whole sample and in patient subgroups with and without diabetes (Table 4). In absence of significant time x group interaction, additional comparison between baseline and post-training levels of glucose and insulin metabolism also showed no improvement in patients with (p = 0.220–0.910) and without diabetes (p = 0.713–0.953) (Table 5). In addition, the exploratory analysis of associations between post-training difference in glucose levels and post-training difference in statin dose showed only significant positive correlation following HL-RT (Spearman`s correlation coefficient = 0.471, p = 0.049) (Additional file 1: Table S3).

**Table 4:** Correlations between post-training difference in body composition and blood markers

			Insulin difference	Glucose difference	HOMA-IR difference	Total cholesterol difference	HDL difference	LDL difference	Triglycerides difference
Non-diabetic (n = 49)	Fat mass difference	Spearman rho	− 0.006	0.067	− 0.016	0.071	0.046	0.032	0.000
		p	0.969	0.645	0.915	0.630	0.752	0.825	1.000
	Lean mass difference	Spearman rho	0.007	− 0.063	0.017	− 0.076	− 0.048	− 0.039	− 0.005
		p	0.963	0.666	0.908	0.603	0.744	0.793	0.973
Diabetic (n = 9)	Fat mass difference	Spearman rho	− 0.233	0.250	− 0.133	0.008	− 0.420	− 0.043	0.417
		p	0.546	0.516	0.732	0.983	0.260	0.913	0.265
	Lean mass difference	Spearman rho	0.233	− 0.250	0.133	− 0.008	0.420	0.043	− 0.417
		p	0.546	0.516	0.732	0.983	0.260	0.913	0.265
Sample (n = 58)	Fat mass difference	Spearman rho	− 0.022	0.149	− 0.016	− 0.006	− 0.038	− 0.046	0.090
		p	0.868	0.264	0.903	0.965	0.775	0.733	0.503
	Lean mass difference	Spearman rho	0.025	− 0.146	0.019	0.002	0.037	0.041	− 0.092
		p	0.855	0.275	0.888	0.988	0.780	0.758	0.491

*Spearman rho* Spearman correlation coefficient, *HOMA-IR* homeostatic model assessment for insulin resistance, *HDL* high density lipoprotein, *LDL* low density lipoprotein

**Table 5:** Baseline and post-training glucose and insulin metabolism in coronary disease patients with and without diabetes

		Baseline	Post-training	t	p
Non-diabetic patients	Glucose (mmol/l)	5.7 (0.8)	5.7. (0.9)	− 0.370	0.713
	Insulin (pmol/l)	69 (48, 116)	73 (47, 111)	− **0.065**	**0.953**
	HOMA2-IR (unit)	1.34 (0.93, 2.19)	1.34 (0.89, 2.13)	− **0.121**	**0.909**
Diabetic patients	Glucose (mmol/l)	8.6 (3.8)	7.2 (2.0)	1.330	0.220
	Insulin (pmol/l)	101 (80, 173)	111 (76, 461)	− **0.533**	**0.652**
	HOMA2-IR (unit)	2.00 (1.70, 4.25)	2.38 (1.46, 8.09)	− **0.178**	**0.910**

Data are presented as mean (standard deviation) or as median (first quartile, third quartile). *HOMA2-IR* homeostatic model assessment for insulin resistance, t-test statistic of paired samples t-test or Wilcoxon rank test (bold text)

## Discussion

This study is one of the first to compare the dose-dependent relationship between RT load and improvements in insulin resistance and lipids profile in patients with CAD enrolled in early phase II CR. The addition of RT to AT, regardless of the RT load showed no additional benefits on insulin resistance and lipids. However, HL-RT and AT decreased total cholesterol and LDL following training intervention, whereas there were no differences between training modalities in body composition or blood biomarkers. In addition, there was also no relationship between post-training difference in body composition and post-training difference in blood markers in patients with CAD.

The levels of insulin resistance were not improved with the addition of RT to AT, likely due to lower HOMA2-IR values than are cut-off values for determining potential metabolic risk in nondiabetic individuals (HOMA2-IR > 1.8) [26]. This contrasts with previous studies, which mostly included patients with obesity, the metabolic syndrome and diabetes with worse metabolic and body composition status (e.g., higher body fat % and body mass index) in comparison to our sample of patients with CAD [27–32]. Moreover, fewer multiple exercise interventions were performed in patients with CAD [11–13, 33, 34]. After exercise-based CR, studies showed no difference between combined AT and RT, and AT, RT or usual care alone in glucose, insulin resistance and/or blood lipids, similarly, as observed in our study. Most interventions with longer training duration (> 8 weeks), regardless of training modality (AT, RT or combined AT and RT), improved insulin resistance and blood lipids levels [33, 34], which partially corroborates with benefits observed following AT and HL-RT in our study. Otherwise, shorter training intervention (< 6 weeks) failed to elicit any between-group or within groups improvements [12, 13], despite using similar RT loads as longer training interventions (60%-65% of 1-RM). In our study, the use of only single lower limb resistance exercise likely elicited suboptimal stimulus for any additional cardiometabolic benefits.

With a high prevalence of co-exiting diabetes in patients with CAD [8], our findings can be compared with similar interventions in patients with metabolic syndrome, prediabetes or diabetes. The studies that enrolled similarly aged patients with metabolic syndrome have demonstrated absence of between-group difference and only post-training improvements in insulin resistance and/or blood lipids following each training modality [27, 28]. On contrary, one study has shown superiority of combined RT and AT over AT alone on insulin resistance in middle-aged obese individuals [30]. When compared with our findings, the authors measured insulin resistance only 12 h after the last training session, which may in combination with more metabolically demanding protocols of AT (weekly exergy expenditure of 14 kcal/kg of body weight) and HL-RT (multiple whole body resistance exercises) explain the discrepancy between studies. In addition, the worse metabolic clinical status of the participants cannot be ruled out [30], as our patients entered the CR with optimized drug therapy and with lower prevalence of diabetes as expected, thus, the improvement in insulin resistance was harder to achieve, regardless of the training modality. Furthermore, our findings are also in line with a recent meta-analysis of patients with type II diabetes that showed similar effects of LL-RT and HL-RT when compared with AT on glycated hemoglobin, insulin levels and insulin resistance [32]. In contrast to our findings, the analysis even showed a superior effects of HL-RT over usual care in reduction of fasting glucose (− 0.92 mmol/l). In addition, LL-RT was associated with a greater decrease in insulin levels than HL-RT when compared to usual care [32], which suggests that gains in muscle endurance may play a superior role over maximal muscle strength gains in improvement of insulin metabolism. However, direct comparison with our study cannot be made due to only indirect comparison between HL-RT and LL-RT in the meta-analysis [32] and due to lower prevalence of diabetes in our study (15%). The effects of dose-dependent relationship between RT load and improvement in insulin resistance also remains to be established in similarly aged older adults without type II diabetes, whereas previous interventions have only combined LL- to moderate load-RT with AT and have showed an improvement in insulin resistance over AT alone [35, 36].

The effect of combined AT and RT on body mass differed among previous studies in patients with CAD [21, 33, 37, 38], with two similarly designed studies supporting our findings [21, 37]. Most previous interventional studies showed decreased fat mass and/or fat % following combined AT and RT [33], which was superior to AT alone [21, 37–39]. In contrast, we have demonstrated an increase in fat % following AT, and maintained fat % following both LL-RT and HL-RT. Our results can be partially explained by an increased energy demands when participating in RT, as similarly increased metabolism after RT was observed in healthy older adults (up to 15% of total daily energy expenditure) [40]. Furthermore, muscle hypertrophy was evident following most combined AT and RT interventions in patients with CAD [21, 37–39], with greater stimulus achieved following moderate-to HL-RT [21] or HL-RT [38] and after longer intervention (> 24 weeks) [21, 37]. Since most of the previous studies applied multiple RT exercises for upper and lower extremities [21, 37, 38], it seems that using only one lower extremity RT exercise may have provided inadequate stimulus to promote superior effects on lean body mass when compared with solely AT. In addition, the discrepancies between the findings can also be attributed to type of measure, as most of the previous studies measured body composition with Dual-energy X-ray absorptiometry, which is more accurate than bioimpedance [41] used in our study.

Our findings, although novel, need to be interpreted with regards to following limitations. Firstly, our study was primarily powered only for primary study outcomes (maximal aerobic capacity and maximal voluntary contraction) [14], therefore all secondary outcomes of this study must be interpreted as exploratory, especially HOMA2-IR. Nevertheless, our sample size was comparable to some of the previous studies in patients with CAD [13, 33]. Secondly, coronavirus-19 pandemic restriction prevented blinding of the outcome assessors. In addition, the staff relocations to other departments also limited the inclusion of more than one lower limb exercise (e.g., leg press) in RT, which may elicit greater changes in body composition, and glucose and lipids metabolism, and consequently distinguished the effects between training interventions. Thirdly, the prevalence of diabetes was low in our sample (15%) compared with recent EUROASPIRE IV survey cohort [8], therefore, our results cannot be directly translated in CAD patients and predominately co-exiting diabetes. Future multimodal training intervention should therefore include more CAD patients with metabolic syndrome or with diabetes. Lastly, higher doses of lipid lowering drugs were likely superior over exercise training effects.

## Conclusions

In conclusion, the combination of RT with AT, regardless of RT load, does not enhance benefits on HOMA2-IR and lipids when compared with solely AT. Therefore, AT alone with a combination of optimal pharmacological therapy and lifestyle modifications (dietary and physical activity advice) presents an adequate training modality to optimally control insulin resistance and blood metabolism. Otherwise, the addition of HL-RT or LL-RT to AT may still provide greater benefits on maximal muscle strength and physical performance over AT alone, as shown in our previous reports [14, 42], and should be therefore applied according to patients` abilities. Despite favorable implications of our RT protocols, future training interventions should include more patients with CAD and diabetes and apply multiple upper and lower limb RT exercise to examine whether greater training workloads would elicit additional benefits on metabolic control compared to AT alone in patients with CAD.

## Supplementary Information

**Additional file 1: Table S1**. Statin or ezetimibe dose at baseline and post-training. **Table S2 **. Lipids lowering drugs at baseline and post-training. **Table S3**. Correlations between post-training difference in glucose levels and statin dose.

## Data Availability

The datasets used and/or analyzed during the current study are available from the corresponding author on reasonable request.

## Abbreviations

ANCOVA: Analysis of covariance
ANOVA: Analysis of variance
AT: Aerobic training
CAD: Coronary artery disease
CR: Cardiac rehabilitation
HDL: High density lipoprotein
HL: High load
HOMA-IR: Homeostatic assessments of insulin resistance
LDL: Low density lipoprotein
LL: Low-load
RT: Resistance training
1-RM: One repetition maximum

## Footnotes

1 https://www.dtu.ox.ac.uk/homacalculator/index.php.
