# Supplementary material for: Insulin resistance, lipids and body composition in patients with coronary artery disease after combined aerobic training and resistance training: a randomised, controlled trial
Source: Diabetol Metab Syndr. 2023 Mar 15;15:47. doi: 10.1186/s13098-023-01017-w (PMC10014406; doi:10.1186/s13098-023-01017-w)
Supplement: Supplementary file 1 — Additional file 1: Table S1. Statin or ezetimibe dose at baseline and post-training. Table S2 . Lipids lowering drugs at baseline and post-training. Table S3. Correlations between post-training difference in glucose levels and statin dose. [file 13098_2023_1017_MOESM1_ESM.docx]

# Additional file Materials

**Table S1.** Statin or ezetimibe dose at baseline and post-training

|  | | N | Baseline | Post-training | 2-way ANOVA/**ANCOVA** | |
| --- | --- | --- | --- | --- | --- | --- |
|  |  |  |  |  | Time effect | Interaction |
| Statin or ezetimibe dose (mg) | AT | 19 | 30 (11) | 37 (8) | p < 0.001 η² = 0.339 | p = 0.496 η² = 0.027 |
|  | LL-RT | 19 | 27 (11) | 34 (10) |  |  |
|  | HL-RT | 21 | 27 (11) | 38 (14) |  |  |
| Data are presented as mean (standard deviation). AT - aerobic training; LL-RT - low-load resistance training; HL-RT - high-load resistance training; ANOVA - analysis of variance; η² - partial eta squared (effect size). Five patients were excluded from the analysis due to change in type of statin. | | | | | | |

**Table S2.** Lipids lowering drugs at baseline and post-training

|  | | Baseline | | | Post-training | | | Total |
| --- | --- | --- | --- | --- | --- | --- | --- | --- |
|  |  | statin or ezetimibe | statin combined with ezetimibe | statin or ezetimibe combined with PCSK-9 inhibitor | statin or ezetimibe | statin combined with ezetimibe | statin or ezetimibe combined with PCSK-9 inhibitor |  |
| AT | f (%) | 18 (95%) | 1 (5%) | 0 (0%) | 9 (47%) | 8 (42%) | 2 (11%) | 19 (100%) |
| LL-RT | f (%) | 18 (95%) | 1 (5%) | 0 (0%) | 11 (58%) | 8 (42%) | 0 (0%) | 19 (100%) |
| HL-RT | f (%) | 20 (95%) | 0 (0%) | 1 (5%) | 14 (67%) | 5 (24%) | 2 (10%) | 21 (100%) |
| Total | f (%) | 56 (95%) | 2 (3%) | 1 (2%) | 34 (58%) | 21 (36%) | 4 (7%) | 59 (100%) |
| Data are presented as mean (standard deviation). AT - aerobic training; LL-RT - low-load resistance training; HL-RT - high-load resistance training; PCSK-9- Proprotein convertase subtilisin/kexin type 9. | | | | | | | | |

**Table S3.** Correlations between post-training difference in glucose levels and statin dose

|  |  | | Post-training change in glucose levels |
| --- | --- | --- | --- |
| Post-training change in statin dose | AT | Spearman`s rho | 0.140 |
|  |  | p (rho) | 0.568 |
|  |  | n | 19 |
|  | LL-RT | Spearman`s rho | 0.068 |
|  |  | p (rho) | 0.788 |
|  |  | n | 18 |
|  | HL-RT | Spearman`s rho | 0.471 |
|  |  | p (rho) | 0.049 |
|  |  | n | 18 |
| rho - correlation coefficient; AT - aerobic training; LL-RT - low-load resistance training; HL-RT - high-load resistance training. Overall, five patients were excluded from the analysis due to change in type of statin. Post-training change = post-training value - baseline value | | | |
